# *Limosilactobacillus fermentum* SLAM 216-Derived Extracellular Vesicles Promote Intestinal Maturation in Mouse Organoid Models

**DOI:** 10.4014/jmb.2405.05028

**Published:** 2024-08-09

**Authors:** Hyejin Choi, Min-Jin Kwak, An Na Kang, Daye Mun, Suengwon Lee, Mi Ri Park, Sangnam Oh, Younghoon Kim

**Affiliations:** 1 Department of Agricultural Biotechnology and Research Institute of Agriculture and Life Science, Seoul National University, Seoul 08826, Republic of Korea; 2 Department of Pharmacology and Systems Physiology, University of Cincinnati College of Medicine, Cincinnati, OH 45267, USA; 3 Korea Food Research Institute, Wanju 55365, Republic of Korea; 4 Department of Functional Food and Biotechnology, Jeonju University, Jeonju 55069, Republic of Korea

**Keywords:** Probiotics, extracellular vesicle, organoid, intestinal maturation, biotherapeutic agents

## Abstract

Probiotics, when consumed in adequate amounts, can promote the health of the host and beneficially modulate the host’s immunity. Particularly during the host’s early life, the gut intestine undergoes a period of epithelial maturation in which epithelial cells organize into specific crypt and villus structures. This process can be mediated by the gut microbiota. Recent studies have reported that the administration of probiotics can further promote intestinal maturation in the neonatal intestine. Therefore, in this study, we investigated the effects of extracellular vesicles derived from the *Limosilactobacillus fermentum* SLAM 216 strain, which is an established probiotic with known immune and anti-aging effects on intestinal epithelial maturation and homeostasis, using mouse small intestinal organoids. As per our findings, treatment with *L. fermentum* SLAM 216-derived LF216EV (LF216EV) has significantly increased the bud number and size of organoid buds. Furthermore, extracellular vesicle (EV) treatment upregulated the expression of maturation-related genes, including *Ascl2*, *Ephb2*, *Lgr5*, and *Sox9*. Tight junctions are known to have an important role in the intestinal immune barrier, and EV treatment has significantly increased the expression of genes associated with tight junctions, such as *Claudin*, *Muc2*, *Occludin*, and *Zo-1*, indicating that it can promote intestinal development. This was supported by RNA sequencing, which revealed the upregulation of genes associated with cAMP-mediated signaling, which is known to regulate cellular processes including cell differentiation. Additionally, organoids exposed to LF216EV exhibited upregulation of genes associated with maintaining brain memory and neurotransmission, suggesting possible future functional implications.

## Introduction

For the past few years, organoids have become a popular tool in conducting biological studies concerning development, biological processes, and interactions with various substances [1]. Historically, investigations focused on the intestine have employed cell and animal models; however, these approaches are limited to the structural, functional, and mechanical aspects of the gut [2]. Therefore, the requirements for laboratory-based biomimetic models have been emphasized to comprehensively delineate gut development and diseases [3, 4]. Given this perspective, intestinal organoids might a suitable model for diverse functionalities, such as the establishment of vital immune barriers to facilitate the absorption of nutrients and water from the environment [5]. Additionally, the self-organized three-dimensional structure of the gut organoid specifically mimics the intestinal structure, reflecting in detail the complex structure of the real intestine and its biological roles [6]. The intestinal organoid model is described as a highly organized self-renewing tissue with proliferative crypts and differentiated villi, and it could continuously differentiate into various intestinal epithelial cell types, including Paneth cells, goblet cells, enteroendocrine cells, and enterocytes. This model could preserve cellular diversities in the gut environment by maintaining the basic physiological functions of the intestinal epithelium [5, 7].

Recent studies have utilized the development of the infant’s gut, along with various diseases including inflammatory bowel disease (IBD), as a diverse disease model [8]. Furthermore, it has been reported that the in vitro maturation process of intestinal organoids closely resembles the maturation of intestinal epithelium *in vivo*; this allows for a detailed investigation of the molecular mechanisms underlying host intestinal epithelial maturation [4, 9, 10]. The maturation of the gastrointestinal tract, particularly during the host’s early stage, plays a pivotal role in shaping the development and maturation of the gut microbiota [11, 12]. This process holds critical significance as it has the potential to impact the optimal formation of the immune system and the sustained well-being of the individual [9, 13, 14]. Hence, the organoid model has proven capable of effectively mimicking the host’s intestinal maturation process, serving as a viable alternative to track this developmental progression during the early phases of the host’s life [4, 8].

Extracellular vesicles with nano-sized particles are released by live organisms, including eukaryotes, bacteria, and archaea [15]. With increasing recognition of the functional roles of microorganisms in animal health and disease, bacterial extracellular vesicles have attracted significant attention among researchers for its functional roles of interactions with hosts, modulation of host immunity, shuttle genetic material, and nutrient delivery within microbial communities [16-19]. LF216EV can exhibit stability under physiological conditions in animals, and they can also deliver various biological molecules such as proteins, enzymes, DNA, RNA, peptidoglycans, and lipids to the host [20]. The delivery-specific features of LF216EV can be applied efficiently to convey functional substances to the host, enhancing host health and investigating therapeutic and diagnostic applications for various diseases [21]. Therefore, this study has investigated the impact of LF216EV from probiotics on the intestinal maturation of the intestinal organoids; we also evaluated it as a suitable in vitro model for assessing the candidate substances on gut development.

## Materials and Methods

### Isolation of Bacterial Extracellular Vesicles

The isolation of *L. fermentum* SLAM 216-derived extracellular vesicles (LF216EV) was performed using the polyethylene glycol-6000 (PEG-6000) method, which was already described in previous studies, with some modifications [22]. In brief, bacterial supernatant was centrifuged at 7,000 ×*g* at 4°C for 30 min to remove any remaining debris and cells. Then, the supernatant was passed through a 0.45 μm filter and a 0.22 μm filter to remove any residual bacterial cell debris. Thereafter, the supernatant was mixed with PEG-6000 (Sigma, USA) to reach a final concentration of 15%; it was then incubated overnight at 4°C. Using a centrifuge, the mixture was centrifuged at 8,000 ×*g* at 4°C for 30 min, and the pelletized LF216EVs were collected, resuspended in phosphate-buffered saline (PBS), and filtered using a 0.22 μm filter [23]. The LF216EVs were kept at −80°C before being used for experiments.

### Characterization of LF216EV

To characterize LF216EV, we have conducted transmission electron microscopy (TEM) and nanoparticle tracking analysis (NTA). TEM investigations were carried out following previously described methods [24, 25]. Specifically, 5 μl of the EV sample was deposited onto a carbon-coated grid; it was allowed to settle for 60 seconds for stabilization. Subsequently, the samples were negatively stained with 2% uranyl acetate and visualized using TEM at NICEM (National Instrumentation Center for Environmental Management, Seoul National University, Republic of Korea). EV size distribution and abundance were assessed using NTA, with a NanoSight NS300 instrument from Malvern. The acquired data were analyzed using NTA software version 3.4.

### Isolation and Culture of Mouse Intestinal Organoids

Mouse jejunal enteroids were generated using a well-established method [26, 27]. In summary, mouse jejunum segments (3-4 inches) were flushed with ice-cold PBS to clean and remove waste. Then, tissue was cut into three pieces and was opened using scissors to expose the villi, which were gently removed using glass slides. After the tissue was washed in ice-cold PBS and chelation buffer, the separated crypts were collected, pelleted, and resuspended in advanced Dulbecco’s modified Eagle’s medium (DMEM, Gibco, USA). The suspended crypts were passed through a 70 mm cell strainer and plated onto a 24-well cell culture plate with 40 μl of Matrigel (Corning, Inc., USA); these were then polymerized and incubated with 400μl of IntestiCult mouse organoid growth medium (STEMCELL Technologies Inc., Canada). The medium was replaced every 2–3 days, and passaging was performed using a 26G syringe after 6–7 days. This study was approved by the Animal Care Committee of Seoul National University (SNU-230612-4).

### Organoid Maturation Assay

For the in vitro maturation assay of organoids, they were harvested from the Matrigel dome, and any remaining Matrigel was removed using ice-cold PBS. For passaging, organoids underwent trypsin–EDTA treatment and were then dissociated in a water bath at 37°C for 5 min. Advanced DMEM was added to halt the dissociation process, followed by centrifugation at 300 ×*g* for 5 min. The separated organoid pellets were resuspended in Matrigel and incubated with IntestiCult mouse organoid growth medium containing LF216EV for 7 days. Representative images of the cultures were taken from the same well and the same field on subsequent days of culture. Additionally, organoid cultures were monitored over time with a snapshot interval of 1 day, spanning several days of culture. Images for determining organoid size and the number of buds were processed and analyzed using ImageJ software, (National Institutes of Health, USA).

### Immunofluorescence Assays

The expression of Ki67 in mouse organoids after incubation with LF216EV for 7 days was examined using immunofluorescence assays [26]. For fixation, organoid pellets were collected in microtubes, and Matrigel was removed with ice-cold PBS. They were then treated with 4% paraformaldehyde for 15 min and permeabilized with 0.5% Triton X-100 in PBS for 20 min at room temperature. Next, the organoids were blocked in 2% donkey serum in 0.01% Triton X-100 in PBS overnight at 4°C. Then, the primary antibody Ki67 (ABclonal, China) was applied to the organoids at 4°C overnight. Afterward, the samples were incubated with Alexa Fluor 488-conjugated goat anti-rabbit IgG (ABclonal) for 1 h at room temperature. Nuclei were stained with DAPI for 5 min. Image acquisition was performed using a confocal microscope.

### Organoid RNA Isolation

For quantitative real-time polymerase chain reaction (RT-qPCR) and transcriptome analysis, organoids were exposed to LF216EV for 24 h. Organoids were harvested in TRIzol reagent (Ambion, USA) for RNA isolation [28]. Then, the total RNA was isolated using the RNeasy Mini Kit (Qiagen, Germany), according to manufacturer’s instructions. Total RNA quality and quantification were measured using a SpectraMax^®^ ABS Plus Microplate Reader (Molecular Devices, USA).

### Quantitative Real-Time Polymerase Chain Reaction Analysis

For the RT-qPCR assay, 1 μg of total RNA was reverse-transcribed into cDNA using the iScript cDNA Synthesis Kit (Bio-Rad Laboratories, USA), and aliquots of synthesized cDNAs were stored at −20°C. Then, 2.5 μl of cDNA was mixed with 10 μl of the SsoAdvanced Universal SYBR Green Supermix (Bio-Rad Laboratories), 1 μl each of the 10 pmol forward and reverse primers, and 7.5 μl of nuclease-free water. RT-qPCR was performed using the Bio-Rad CFX96 Real-time PCR System (Bio-Rad Laboratories) with target gene primers. The cycling conditions included polymerase activation and DNA denaturation at 95°C for 30 sec, followed by 40 cycles of 95°C for 15 sec and annealing at 60°C for 30 sec. Subsequently, melt curve analysis was performed at 65°C with 5 sec/step. The gene expression of each sample was normalized using GAPDH. The relative fold change in gene expression was calculated using the 2ˆ(–ΔΔCT) method [29]. The PCR primers used in the qRT-PCR analysis were listed in Table 1 [30].

### Transcriptome Analysis

In this study, transcriptome sequencing of organoids was conducted to obtain gene expression values, analyze differentially expressed genes, and conduct functional classification and gene annotation based on gene ontology and pathway information for significant genes [31, 32]. Total RNA concentration was determined using the Quant-it RiboGreen RNA Assay Kit (Invitrogen, USA) with the VICTOR Nivo Multimode Microplate Reader (PerkinElmer, USA); meanwhile, RNA integrity was assessed using an Agilent Technologies 2100 Bioanalyzer (Agilent Technologies, USA). Only high-quality RNA samples were utilized for RNA library construction. Libraries of EV-treated and non-treated organoids were prepared using the Illumina TruSeq Stranded Total RNA Library Prep Gold Kit (Illumina, Inc., USA), according to the manufacturer’s protocol. Following purification and enrichment with PCR, the final cDNA libraries were sequenced on an Illumina NovaSeq platform [33].

### Statistical Analysis

Each experiment was replicated at least three times, and results are expressed as mean ± standard error (SEM). Statistical analysis involved one-way analysis of variance (ANOVA), followed by Tukey’s post-hoc test, conducted using the GraphPad Prism software 10.0. Significance levels were denoted as *p < 0.05, **p < 0.01, and ***p < 0.001, indicating significant differences across all replicates.

## Results

### Characterization of *L. fermentum*-Derived Extracellular Vesicles

**Fig. 1:**
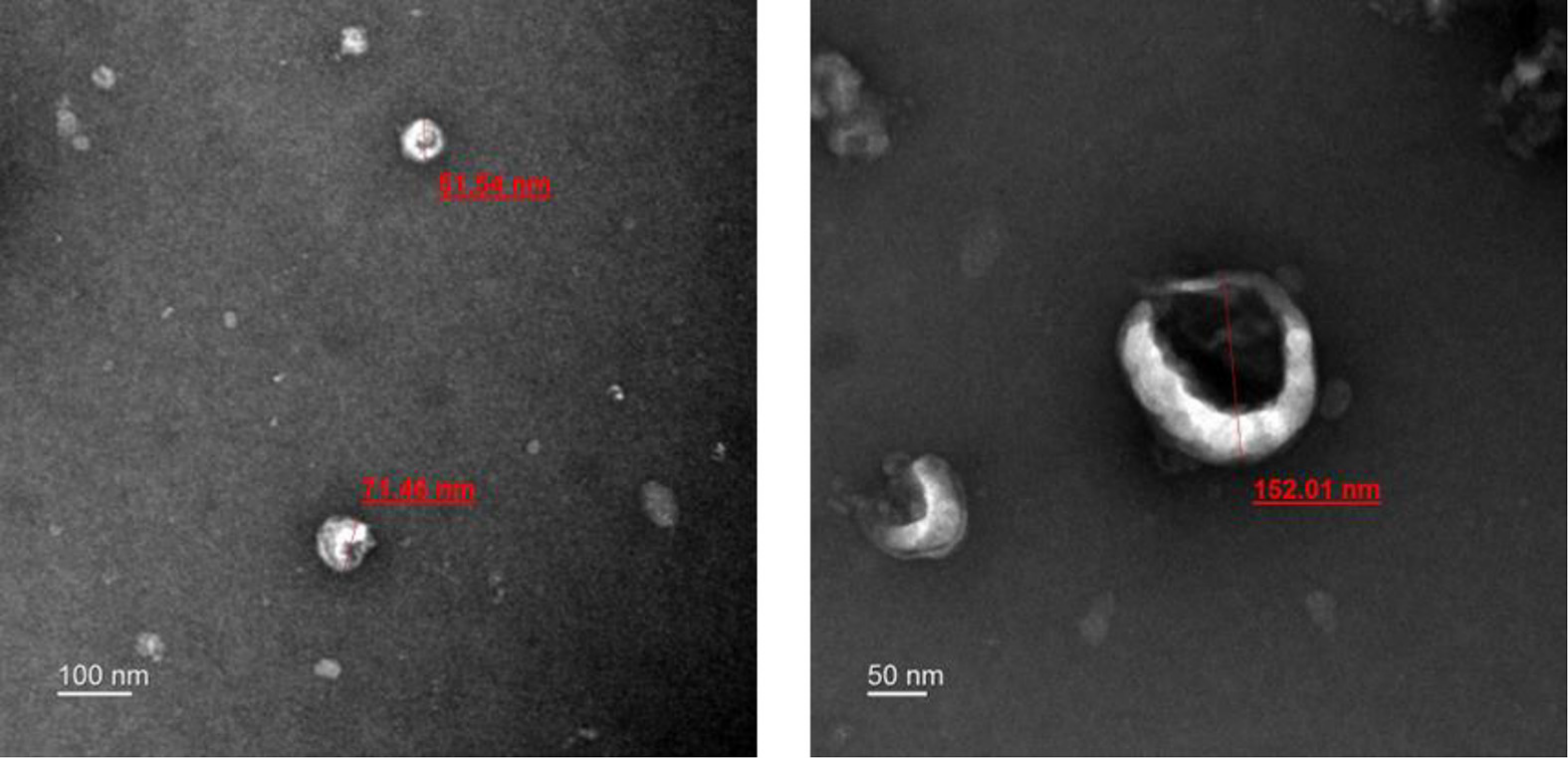
Identification of LF216EV by TEM analysis. The LF216EV particle is shown in the TEM image. Scale bars, 100 nm (left) and 50 nm (rignt).

Extracellular vesicles derived by *L. fermentum* SLAM 216 (LF216EV) were isolated through a polyethylene glycol-based method. The identification of LF216EV was accomplished by transmission electron microscopy (TEM), which is a widely acknowledged visualization technique, and confirmed the typical appearance of well-known LF216EV (Fig. 1). Subsequent analysis of EV size and number concentration was conducted using nanoparticle tracking analysis (NTA). The average size of LF216EV was determined to be 115.0 nm (unpublished data).

### LF216EV Improves Mouse Intestinal Organoid Maturation

**Fig. 2:**
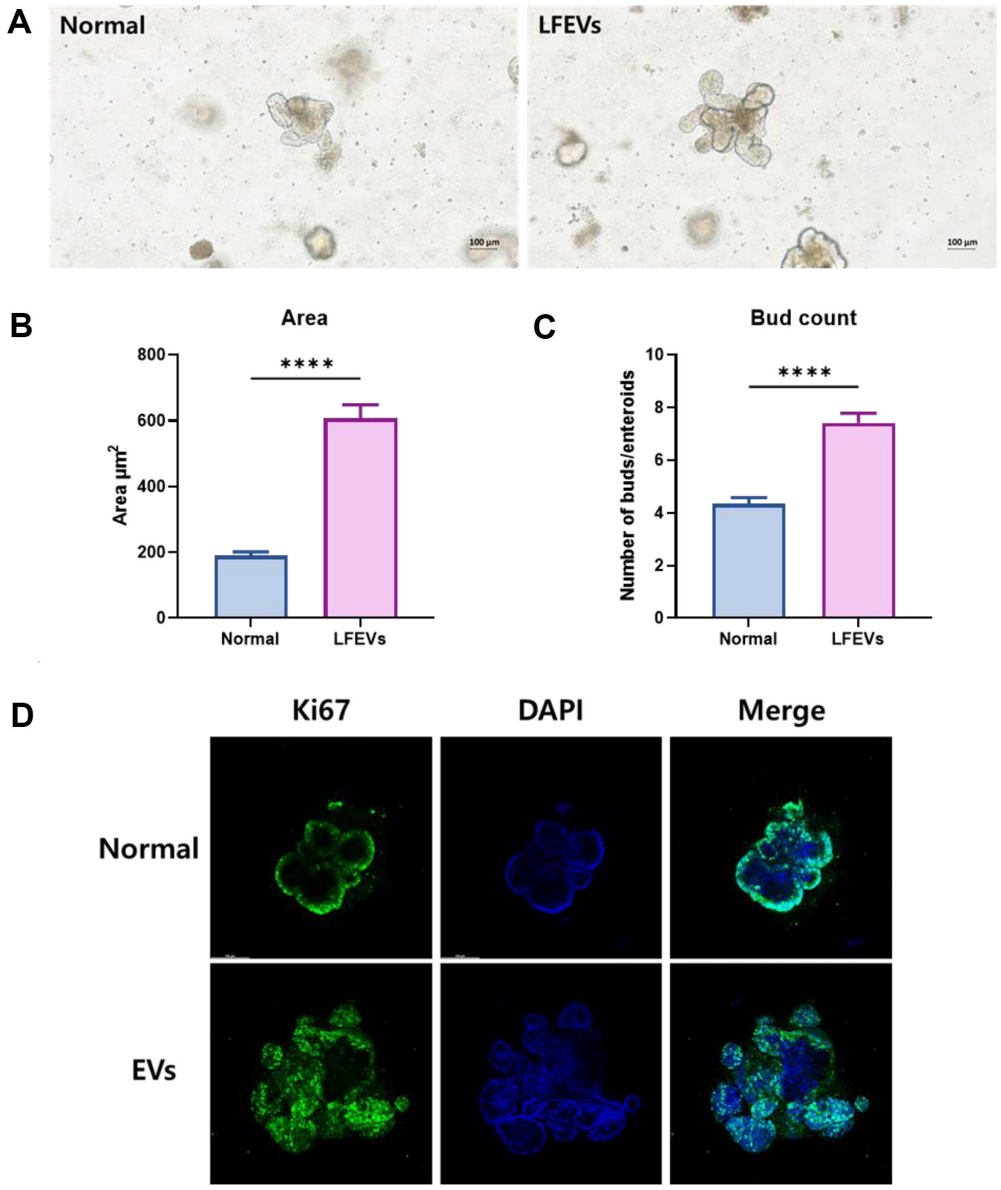
LF216EV promotes the development of mouse intestinal organoids. (**A**) Representative images showing mouse intestinal organoids cultured with LF216EV in day 7. (**B**) Organoid area after 7 days culture with LF216EV. (**C**) Number of organoid bud per organoids after a 7-day culture with LF216EV. LF216EV, *L. fermentum* SLAM 216-derived LF216EV. *=*p*<0.05, **=*p*<0.01, ***= *p*<0.001, normal control vs. LF216EV. (**D**) Protein expression by immunofluorescence analysis for maturation marker KI67 of intestinal organoids.

As per our investigation on the effects of LF216EV on the host gut, we conducted experiments utilizing mouse intestinal organoids as a model system. Organoids cultured with LF216EV exhibited accelerated growth as compared to organoids cultured under non-treated conditions, resulting in a more robust morphology after a 7-day culture period (Fig. 2A). Organoids incubated with concentrations of LF216EV for 7 days exhibited a significant increase in terms of size as compared to non-treated organoids (Fig. 2B). Also, treatment with LF216EV significantly increases the number of buds (Fig. 2C). Epithelial development was examined using immunofluorescence staining, and KI67, which is a marker of epithelial proliferative activity, was found to be highly concentrated in organoids exposed to LF216EV (Fig. 2D).

**Fig. 3:**
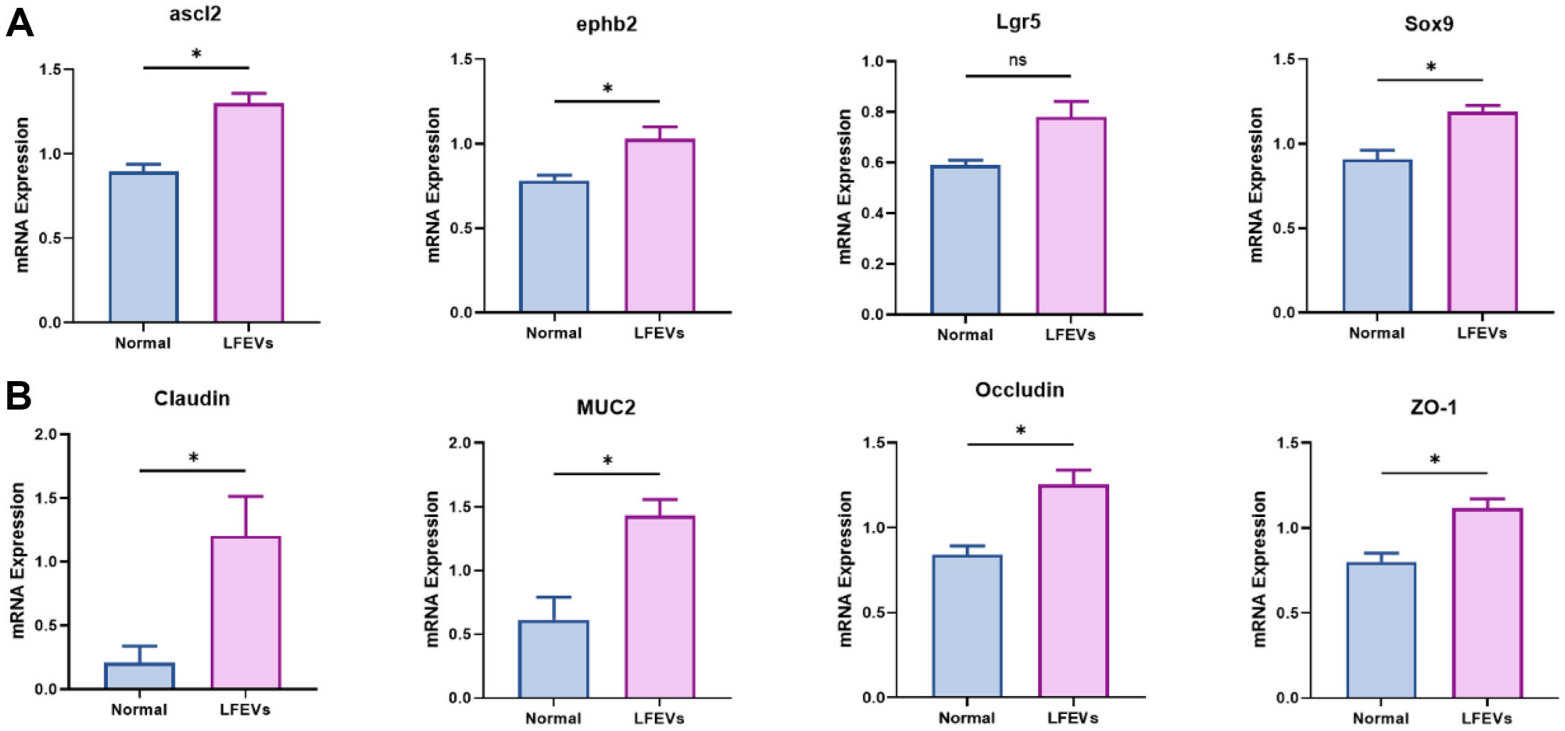
Quantitative RT-PCR analysis of maturation and gut tight junction markers in LF216EV-treated organoids. (**A**) Quantitative RT-PCR analysis for markers of maturation in LF216EV-treated organoids. (**B**) Quantitative RTPCR analysis for markers of gut tight junction in LF216EV-treated organoids. LF216EV, *L. fermentum* SLAM 216-derived LF216EV. *=*p*<0.05, **=*p*<0.01, ***= *p*<0.001, normal control vs. LF216EV.

Hence, we employed RT-qPCR to assess whether the phenotypic alterations induced by LF216EV were reflected at the gene level (Fig. 3). Organoids incubated with LF216EV exhibited a significant upregulation in the expression of genes associated with proliferation and differentiation (*Ascl2*, *Ephb2*, *Lgr5*, *Sox9*; Fig. 3A). LF216EV-treated organoids showed an approximately 1.5-fold increase in *Ascl2* gene expression increased by approximately 1.5-fold, *Ephb2* gene expression increased by approximately 1.3-fold, *Lgr5* increased by 1.9-fold (10^9^) and 1.3-fold (10^10^), respectively, and *Sox9* increased by approximately 1.3-fold. Moreover, organoids incubated with LF216EV exhibited a notable increase in the expression levels of specific markers closely associated with the integrity of gut tight junctions (Claudin, *Muc2*, *Occludin*, *Zo-1*; Fig. 3B). Expression of the *Claudin* gene increased more than 5-fold in all LF216EV treatments, the *Muc2* gene increased more than 2-fold, *Occludin* increased about 1.5-fold, and *Zo-1* increased about 1.4-fold. These enhancements underscore the potential impact of LF216EV on both structural development and functional aspects of the intestinal barrier within the organoid model system.

### LF216EV-Induced Gene Expression Changes in Mouse Intestinal Organoids

**Fig. 4:**
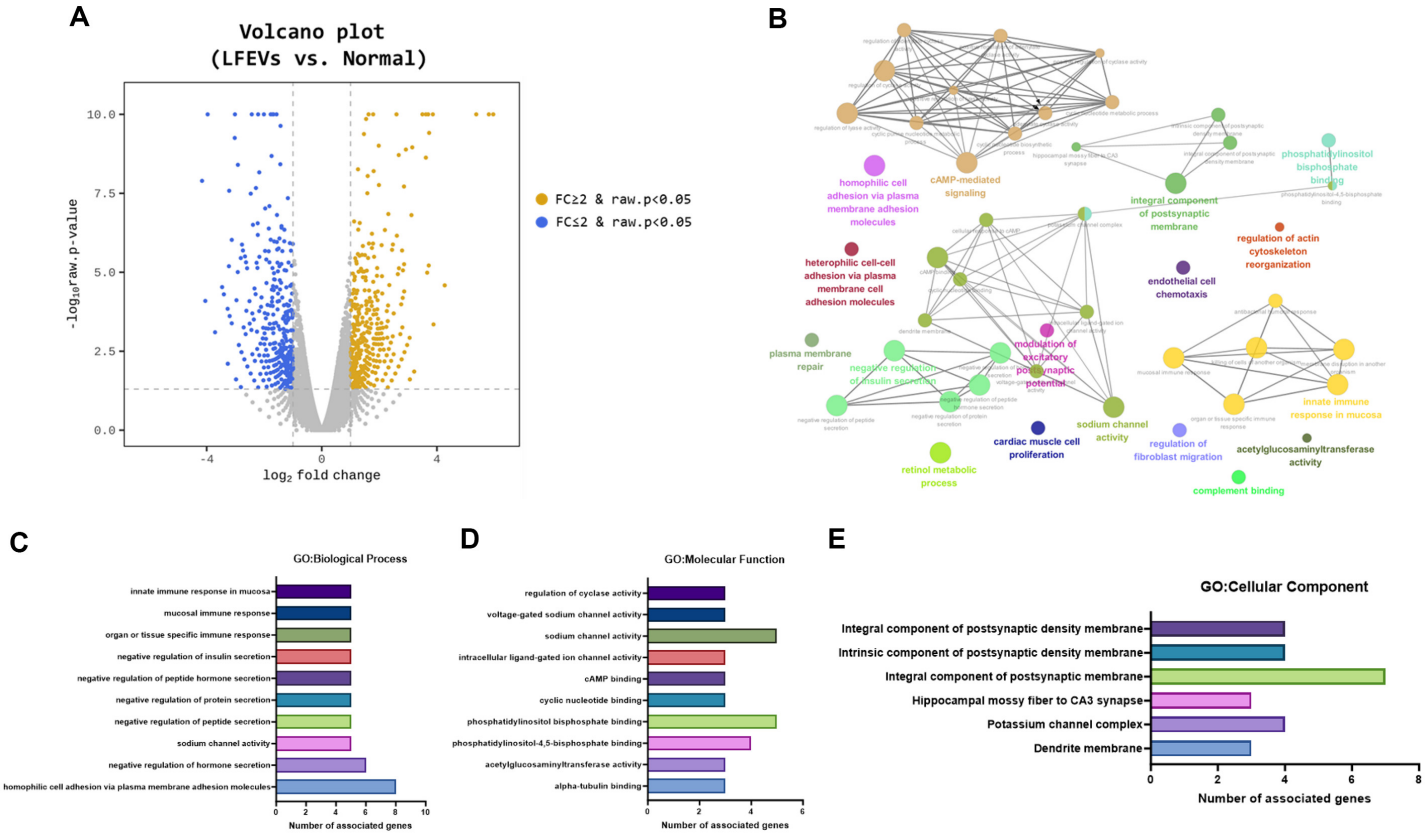
Transcriptional profiling of organoids treated with LF216EV. (**A**) Volcano plot of transcriptome differences. (**B**) Cytoscape pathway network of significantly upregulated genes in LF216EV-treated organoids. (**C**) Gene Ontology (**GO**) enrichment analysis for the term in the biological process category. (**D**) GO enrichment analysis for the term in the molecular function category. (**E**) GO enrichment analysis for the term in the cellular component category. LF216EV, *L. fermentum* SLAM 216-derived LF216EV.

Here, we focused on investigating the genes regulated by LF216EV using RNA sequencing analysis. RNA from organoids exposed to LF216EV (treatment group) and non-exposed organoid RNA were sequenced, wherein the gene expression levels were noted to vary between the LF216EV treatment group and the control group (Fig. 4A). The LF216EV treatment group significantly upregulated 425 genes and downregulated 387 genes as compared to the control group. Increase in genes was associated with integral components of the postsynaptic membrane, cAMP-mediated signaling, and the innate immune response in the mucosa (Fig. 4B). Furthermore, we performed Gene Ontology (GO) analysis on RNA-seq data, and organoids, exposed to LF216EV, showed 301 significantly regulated GO terms (27 terms for biological process (BP), 78 terms for molecular function (MF), and 196 terms for cellular component (CC)). The main GO terms in BP were as follows: GO:0002227 (innate immune response in mucosa), GO:0002385 (mucosal immune response), GO:0002251 (organ- or tissue-specific immune response), GO:0005275 (sodium channel activity), and GO:0007156 (homophilic cell adhesion via plasma membrane adhesion molecules) (Fig. 4C). In MF, the main GO terms were GO:0008375 (acetylglucosaminyltransferase activity), GO:0030551 (cyclic nucleotide binding), GO:0030552 (cAMP binding), GO:0005217 (intracellular ligand-gated ion channel activity), and GO:0031279 (regulation of cyclase activity) (Fig. 4D). Meanwhile, in CC, the main GO terms were GO:0032590 (dendrite membrane), GO:0034705 (potassium channel complex), and GO:0099061 (integral component of postsynaptic density membrane) (Fig. 4E). When organoids were cultured with LF216EV, the top ten genes were significantly increased (Table 1); meanwhile, the expression levels of testis-specific serine kinase 6 (*Tssk6*), trace amine-associated receptor 8C (*Taar8c*), solute carrier family 8-(*Slc8a2*), and small proline-rich protein 2B (*Sprr2b*) related genes were also significantly increased. Among the ten upregulated genes, the genes specifically associated with intestinal maturation are *Slc8a2*, *Sprr2b*, *Gpr135*, *Mamld1*, *B3gnt5*, and *Cd1d1*.

## Discussion

The gastrointestinal tract (GIT) acquires functionality through a process of maturation, and the villi and microvilli features were built in this process by the emergence of various cell types with enhanced structural integrity [34, 35]. The mouse intestinal organoid, which is used in this study, represents a 3D culture model capable of partially simulating phenotypic structure, cellular composition, and intestinal function. Moreover, this organoid model could overcome the limitations of the monolayer model for gut epithelium in a conventional host; it allows for more comprehensive investigations. In particular, the organoid model can reproduce the interactions among various types of cells in the intestine, including Paneth cells, goblet cells, enterocytes, and stem cells, and this novel model could simulate the absorption and secretion capacities of the intestine through the self-organization of multicellular structures and functional cells [36, 37].

In this study, we have investigated the impact of probiotic-derived EV on host intestinal formation and maturation using a mouse intestinal organoid model. LF216EV was found to promote growth; it also increased the number of buds in organoids within the mouse intestine. Additionally, LF216EV significantly enhanced the expression of genes associated with organoid maturation, such as *Ki67*, *Ascl2*, *EphB2*, and *Sox9*, with a significant elevation in the expression of genes crucial for tight junction formation, including *Claudin*, *Muc2*, *Occludin*, and *Zo-1*. These findings strongly suggest that LF216EV plays a robust role in promoting intestinal barrier integrity and development. The early stages of the host’s life are critical for intestinal maturation, which, in turn, promotes the formation of a mature gut microbiota community; moreover, it has been essential for the long-term health of the host [38, 39].

Particularly, the homeostasis of the gut relies significantly on maintaining the physical barrier of the intestinal epithelium, which serves as a crucial boundary separating the host from pathogenic bacteria and compounds [40, 41]. The intestinal barrier heavily relies on cell-to-cell adhesion junctions of the surrounding intestinal epithelial cells (IEC) at the apical surface, and they form a polarized monolayer with distinct apical and basolateral domains [42]. As per RNA sequencing analysis, LF216EV significantly upregulated the genes associated with intestinal epithelial development, such as *GPR135* and SPRR2B, confirming their induction of tight junction formation within the intestine [43, 44]. Furthermore, it was found that LF216EV significantly upregulated the genes directly related to immunity, such as *TAAR8C*, *B3GNT5*, and *CD1D1*, suggesting their potential to regulate the host’s immune response and protect the host from pathogens and external environments [45-47]. Collectively, our findings confirmed the potential of EV-induced mature intestinal barrier adhesion in promoting healthy neonatal gut colonization and immune system maturation.

In a recent research, supplementation of probiotics and probiotic-derived factors in early life can promote host growth and immune development through the establishment of gut microbiota community [11, 48, 49]. Consequently, various efforts were evaluated to support the healthy development of the gastrointestinal microbiota during the early stages of the host’s life. For example, various researchers demonstrated that infants who consume formula milk supplemented with probiotics, such as *Lacticaseibacillus rhamnosus*, *Limosilactobacillus reuteri*, and *Bifidobacterium lactis* has a reduced risk of necrotizing enterocolitis with the improvement of stool consistency and infant growth [50-52].

While probiotics are widely available to consumers in various forms, limitations in ensuring the stability of viable microbial counts have been observed due to processing constraints and shelf-life limitations during the production and distribution of commercial products [53]. Therefore, the importance of postbiotics, serving as bacterial metabolites and their components, has been emphasized recently as a means to address these issues [54, 55]. In this study, we introduced a novel form of postbiotics, that is, EV. They are nano-sized particles enveloped by a lipid bilayer that transport cell-derived compounds and are generated through microbial metabolism. These particles can interact with the host by modulating signaling pathways such as metabolic pathways, fatty acid degradation, and biosynthesis of cofactors [56].

Furthermore, postbiotics have been proposed as a safe alternative to using live bacteria to avoid the potential side effects of probiotics in vulnerable hosts such as immunocompromised patients [57]. In 2021, Hao demonstrated that EVs derived from *L. plantarum* Q7 could alleviate ulcerative colitis in mice; meanwhile, Tong and colleagues also proposed that EVs derived from *L. rhamnosus* GG could attenuate the inflammation in dextran sodium sulfate (DSS)-induced colitis mice and regulated the host’s immune response [58, 59]. As we mentioned several studies have investigated the impact of probiotic-derived EVs on disease, but our study is the first to demonstrate that probiotic-derived EVs can promote host maturation of the gut. Accumulated evidence has indicated that bacterial-derived EV could deliver a variety of biomolecules and genetic material to the host. Therefore, studies related to the application of EVs in food and pharmaceutical industries have been continued, because they can function in a non-living state, offer a longer shelf-life with better environmental resilience, and has more stability [60]. Therefore, LF216EV's ability to promote intestinal maturation in the host, which was confirmed in this study, could be utilized as a more effective functional material. LF216EV has the potential to be a more effective functional material when delivered to the host at an early stage with the least amount of side effects, and its stability is ensured until it reaches the host.

In this study, the LF216EV was determined to facilitate the delivery of various biomolecules to the intestinal organoid model, and it could also induce regulated gene expression. Subsequently, KEGG pathway analysis revealed the upregulation of pathways including cAMP-mediated signaling, innate immune response in the mucosa, sodium channel activity, and phosphatidylinositol bisphosphate binding, thereby modulating the host’s immune response. Moreover, the promotion of intestinal maturation was observed, suggesting its potential to create a mature gut microbiome environment.

Collectively, this study demonstrated the potential benefits of supplementing probiotic-derived EVs in the early development of the host, as they promote intestinal maturation and its microbial environment and potentially reduce host diseases by regulating genes related to immunity. However, due to the incomplete elucidation of the physiologically active components and precise mechanisms of LF216EV, the question remains unanswered regarding the specific mechanisms underlying the effects mediated by LF216EV. Therefore, through further research involving the characterization and identification of LF216EV components, a broader understanding of the mechanisms of action of LF216EV can be achieved. Also, this finding provides new insights into the role and functionality of LF216EV as potential postbiotics, thus offering the possibility of serving as effective substances supporting intestinal maturation in the host, potentially replacing probiotics.

## Figures and Tables

**Table 1:** Significantly upregulated genes in LF216EV-treated organoids.

Genes	Description	Fold change LF216EV/Control
*Tssk6*	Testis-specific serine kinase 6	55.88
*Taar8c*	Trace amine-associated receptor 8C	41.25
*Slc8a2*	Solute carrier family 8 (sodium/calcium exchanger), member 2	14.74
*Sprr2b*	Small proline-rich protein 2B	14.51
*Gpr135*	G protein-coupled receptor 135	13.26
*Mamld1*	Mastermind-like domain containing 1	12.65
*Fank1*	Fibronectin type 3 and ankyrin repeat domains 1	12.28
*B3gnt5*	UDP-GlcNAc:betaGal beta-1,3-N-acetylglucosaminyltransferase 5	12.26
*Cd1d1*	CD1d1 antigen	11.29
*Marveld1*	MARVEL (membrane-associating) domain containing 1	7.58
